# Football training as a non-pharmacological treatment of the global aging population—A topical review

**DOI:** 10.3389/fragi.2023.1146058

**Published:** 2023-02-09

**Authors:** Magni Mohr, Ioannis G. Fatouros, Muhammad Asghar, Pasqualina Buono, George P. Nassis, Peter Krustrup

**Affiliations:** ^1^ Department of Sports Science and Clinical Biomechanics, SDU Sport and Health Sciences Cluster (SHSC), University of Southern Denmark, Odense, Denmark; ^2^ Centre of Health Science, Faculty of Health, University of the Faroe Islands, Tórshavn, Faroe Islands; ^3^ Department of Physical Education and Sport Science, University of Thessaly, Trikala, Greece; ^4^ Department of Biology, Lund University, Lund, Sweden; ^5^ Department of Movement Sciences and Wellness, University Parthenope, Naples, Italy; ^6^ CEINGE-Biotecnologie avanzate Francesco Salvatore, Napoli, Italy; ^7^ Department of Physical Education, College of Education, United Arab Emirates, University, Al Ain, Abu Dhabi, United Arab Emirates; ^8^ Danish Institute for Advanced Study (DIAS), University of Southern Denmark, Odense, Denmark; ^9^ Sport and Health Sciences, University of Exeter, Exeter, United Kingdom

**Keywords:** soccer, exercise training, multicomponent training, hypertension, T2D, inflammation, antiageing effects on metabolic health, cancer

## Abstract

In the present topical mini-review, the beneficial impact of small-sided game football training for the increasing elderly global population is presented. As a multicomponent type of physical activity, football training executed on small pitched with 4–6 players in each team is targeting a myriad of physiological systems and causes positive adaptations of relevance for several non-communicable diseases, of which the incidence increases with advancing age. There is strong scientific evidence that this type of football training promotes cardiovascular, metabolic and musculo-skeletal health in elderly individuals. These positive adaptations can prevent cardiovascular disease, type 2 diabetes, sarcopenia and osteoporosis, and lower the risk of falls. Also, football training has been proven an efficient part of the treatment of several patient groups including men with prostate cancer and women after breast cancer. Finally, regular football training has an anti-inflammatory effect and may slow the biological aging. Overall, there is a growing body of evidence suggesting that recreational football training can promote health in the elderly.

## Introduction

A world-wide challenge is the increasing aging population, and in 2040 a total of 14% of the entire world’s population is estimated to be older than 65 years (Christensen et al., 2009). Epidemiological report shows, age as a main player in the risk of non-communicable diseases with higher prevalence of cardiovascular and metabolic diseases, neurological conditions, and musculoskeletal disorders in elderly than their younger counterparts (Christensen et al., 2009). The socio-economic burden of an elevated portion of people suffering from chronic disease and receiving conventional medical treatment may exert a major challenge in the future to come (Christensen et al., 2009). Thus, developing of low-cost, efficient, broad-reaching, and sustainable non-pharmacological treatment strategies for the elderly population are highly warranted.

Strong evidence demonstrates that exercise training is a cornerstone in the treatment of cardiovascular, metabolic, and musculoskeletal diseases (Pedersen and Saltin, 2015) and the application of exercise as medicine is increasing. However, to treat age-related pathological conditions with exercise, healthcare personnel needs treatment protocols that on the one side have a broad-spectrum health impact and on the other side also are motivating, socially beneficial and promote long-term adherence in order to enable sustainable behavioural change in the elderly. Small-sided game (SSG) football training (Milanović et al., 2015; Krustrup et al., 2018a) has been suggested to encompass the right ingrediencies to fulfil these requirements. Firstly, this type of recreational football has been proven to be an intense and versatile combination of high-intensity interval training (HIIT), endurance training and strength training with marked positive results on the cardiovascular, metabolic and musculoskeletal health profile (Milanović et al., 2015; Krustrup et al., 2018a; Milanovic et al., 2019; Milanovic et al., 2022). Secondly, recreational football training has been demonstrated to be a fun and social activity that promotes adherence, due to high flow, fun and intrinsic motivation scores, and low ratings of perceived exercise and worry (Elbe et al., 2010; Ottesen et al., 2010). Despite the vast amount of work done in this field, including several reviews and meta-analysis, no reviews have been published on the effect of SSG football training on elderly populations and healthy aging. In this review, the term SSG and recreational football were used, which covers 4v4-6v6 games on small pitches played for 1 h 2-3 times weekly (Elbe et al., 2010; Randers et al., 2014; Bangsbo et al., 2015; Milanović et al., 2015; Krustrup et al., 2018a).

The objective of the present topical review is to discuss the potential of SSG football training as a broad-spectrum non-pharmacological treatment protocol for elderly. The overview covers the research on SSG football training as treatment of cardiovascular diseases, type 2 diabetes, cancer, inflammation, osteopenia and sarcopenia, as well as the possible impact on biological aging in elderly individuals.

## Effects on cardiovascular health

Cardiovascular disease (CVD) covers several pathological conditions such as cerebrovascular disease, hypertension, coronary heart disease, heart failure and intermittent claudication, all of which exercise is suggested to be a cornerstone in the prevention and treatment (Pedersen and Saltin, 2015). Moreover, CVD, as well as main risk factors such as arterial hypertension, obesity, low cardiorespiratory fitness and hypercholesterolemia are all increasing with advancing age (Pedersen and Saltin, 2015).

Studies using SSG football interventions of several elderly populations including patient groups demonstrate high cardiovascular loading during small-sided game training (Randers et al., 2014; Bangsbo et al., 2015; Krustrup et al., 2018a), which is important to induce broad-spectrum adaptations in cardiovascular health profile. This is further supported by meta-analysis which shows that football training 2-3 times weekly for 3-4 months induces clinically relevant lowering of blood pressure, body fat content, resting heart rate and plasma lipids (Milanovic et al., 2019) and increases maximum oxygen uptake (Milanović et al., 2015; Oja et al., 2015). These beneficial adaptations, which markedly lower the overall CVD risk are also observed in hypertensive middle-aged and elderly men and women (Krustrup et al., 2013; Mohr et al., 2014; Schmidt et al., 2014; Krustrup et al., 2017; Skoradal et al., 2018a). Moreover, cardiac health is improved in football trials in elderly populations (Schmidt et al., 2013; Schmidt et al., 2014).

Collectively, this data suggests that regular SSG football training has great potential as an alternative treatment to patients with cardiovascular disease. On that basis the treatment protocol “Football for the Heart” has been developed as a translational research initiative and sport concept in a close collaboration between researchers at the University of Southern Denmark, The Danish FA and The Heart Foundation in Denmark (for specific information see https://www.sdu.dk/da/om_sdu/institutter_centre/c_isc/forskningsprojekter/projekter/2019_fodbold_for_hjertet).

## Effects on metabolic health, cancer, and inflammation

The pathogenesis of obesity-related diseases, is characterized by chronic, low-grade inflammation (de Heredia et al., 2012; Esser et al., 2014; Saltiel and Olefsky, 2017), recently also called “metaflammation,” i.e., related inflammation (Hotamisligil, 2006). Furthermore, aging is associated with a prolonged accumulation of damage to macromolecules and cell structures that elicit a similar inflammatory response in the circulation and within solid tissues which is involved in the pathophysiology of numerous age-related conditions (Draganidis et al., 2016; Walker et al., 2022). Although a chronic condition, metaflammation engages similar cellular/molecular components of the acute inflammatory response, and compelling evidences are mounted on its role in the pathogenesis of type 2 diabetes mellitus (T2DM) and diabetes-related cardiovascular complications (Hotamisligil, 2017). Metaflammation involves various organs (i.e., skeletal muscle, liver, adipose tissue, brain, heart, pancreas) (Saltiel and Olefsky, 2017) and is associated with hypertrophic adipose tissue, macrophage infiltration in adipose tissue, and a pro-inflammatory phenotype of immune cells that secrete pro-inflammatory adipokines and cytokines that ultimately induce this systemic inflammation (Karczewski et al., 2018; Kawai et al., 2021). Although the mechanisms inducing metaflammation are still unclear, recent reports indicate that inflammasome, multimolecular protein complexes combined in response to pathogen- or danger-associated molecular patterns, stimulation is pivotal (Guo et al., 2015; Christgen and Kanneganti, 2020). Numerous investigations have shown that anti-inflammatory treatments may be therapeutic for obesity and related comorbidities such as T2DM (Zeyda and Stulnig, 2009; Kawai et al., 2021).

Prolonged physical inactivity and increased adiposity predispose to systemic inflammation manifested as elevated levels of pro-inflammatory markers (e.g., CRP, leptin, IL-6, IL-1β, TNF-α) and the onset of lifestyle diseases such as T2DM and cardiovascular disease (Popa et al., 2007; You et al., 2013). In contrast, anti-inflammatory molecules (e.g., adiponectin, IL-10) that enhance insulin sensitivity and endothelial function are suppressed and insulin resistance is increased (Pradhan et al., 2001; Bouassida et al., 2010). Contrarily, chronic physical activity and exercise training attenuate metaflammation (∼30% lower CRP, IL-1β, ∼20% lower TNF-α) and protect against lifestyle diseases (Arita et al., 1999). Systematic aerobic and/or resistance exercise training may improve the inflammatory profile and lower oxidative stress (Fatouros et al., 2004; Panagiotakos et al., 2005; Petersen and Pedersen, 2005; Donges et al., 2010). However, these exercise modalities are characterized by high attrition and low attendance rates (Batrakoulis et al., 2022).

Hybrid-type multicomponent exercise modalities such as SSG football (Elbe et al., 2010; Randers et al., 2014; Bangsbo et al., 2015; Milanović et al., 2015; Krustrup et al., 2018a) are causing broad-spectrum adaptation (Batrakoulis et al., 2022) in a safe environment (Barbosa et al., 2020). SSG training induces substantial improvements of both endurance and muscular strength at the same or even greater extent than aerobic or resistance exercise alone (Krustrup et al., 2010a; Randers et al., 2010; Krustrup et al., 2018a), adaptations that may exert an anti-inflammatory effect (Bouassida et al., 2010). Indeed, both cycling and small-sided football training improved the inflammatory profile by lowering CRP, IL-6, leptin and body fat levels in inactive, middle-aged and elderly men (Mendham et al., 2014; Vorup et al., 2017). Similarly, lifelong SSG training was shown to improve muscle’s oxidative metabolism and DNA repair and improve telomere shortening and senescence indicating that recreational football may represent not only an anti-inflammatory treatment but also an anti-senescence strategy that could potentially prevent inflammaging and promote cardiovascular integrity (Mancini et al., 2017; Hagman et al., 2020; Imperlini et al., 2020). Moreover, recreational football training appears to augment the expression of genes of proteins involved in auto-lysosomal, proteasome-regulated protein degradation mechanism and apoptosis in the aged (Mancini et al., 2019). It is noteworthy that SSG training (4v4-6v6) induced a ∼60% rise of antioxidant enzyme activity in older healthy males (Andersen et al., 2016). This evidence strongly supports that SSG football training may be used as a sustainable potential anti-inflammatory treatment in both middle-aged and older individuals.

## Type 2 diabetes mellitus

T2DM is a lifestyle disease characterized by an impaired insulin release, insulin resistance in various tissues, increased metaflammation, hyperglycaemia, comorbidity (e.g., CVD) increased adiposity and ectopic fat. It is associated with increased morbidity and mortality placing a serious economic burden on healthcare systems and is expected to affect more than 370 million people by 2030 (Shaw et al., 2010; Flores-Le Roux et al., 2011; Yang et al., 2013; Jagannathan and Bergman, 2017). Physical exercise can prevent T2DM and can lower T2DM-associated hyperglycaemia, improves comorbidity and lowers body fat and metaflammation in T2DM patients (Pedersen and Saltin, 2015).

SSG football training, as defined in the introduction, appears to affect the glycemic control of adults with T2DM by reducing glycosylated haemoglobin (HbA1c) (Schmidt et al., 2013; Andersen et al., 2014; de Sousa et al., 2014; de Sousa et al., 2017; Skoradal et al., 2018a), and elicits a considerable fat loss (∼3.5 kg) in elderly with and without pre-diabetes (Randers et al., 2014; Skoradal et al., 2018a) which may be attributed to an increased lipolytic activity as well as improved skeletal muscle mitochondrial and beta oxidation capacity (Krustrup et al., 2010b; Nordsborg et al., 2015; de Sousa et al., 2017). Skeletal muscle’s mitochondrial function characterizes T2DM pathophysiology and seems to be promoted by football training (Bangsbo et al., 2009; Wojtaszewski, 2009; Nordsborg et al., 2015). Furthermore, SSG training has been shown to promote GLUT-4 protein expression which facilitates glucose uptake in skeletal muscle (Mohr et al., 2017).

SSG training also appears to help alleviate the co-morbidity associated with T2DM by improving body composition, inducing favorable effects on the functional and structural characteristics of the cardiac tissue and lowering blood lipids and arterial blood pressure (Krustrup et al., 2010b; Schmidt et al., 2013; Andersen et al., 2014; de Sousa et al., 2014; de Sousa et al., 2017; Skoradal et al., 2018a; Paul et al., 2018; Milanovic et al., 2019; Paul et al., 2019). Also, 12–16 weeks of SSG training twice weekly resulted in a considerable rise of >1 kg of lean body mass in pre-diabetics and T2DM patients (Andersen et al., 2014; Skoradal et al., 2018a) which is crucial because diabetes is associated with a profound loss of muscle mass. (Pedersen and Saltin, 2015). These adaptations may be attributed to a greater IGF-1/IGFBP-3 ratio and lower ammonia levels seen with SSG training suggesting an attenuation of muscle protein catabolism (de Sousa et al., 2014; de Sousa et al., 2017). Elevated muscle mass also provide a greater storage space for glycogen and thus upregulates glucose clearance from the circulation. Moreover, SSG training-induced hypertrophy will upregulate insulin and its blood transport to tissues. Finally, SSG training has a significant impact on physical performance of adults with T2DM. Middle-aged and older adults with T2DM and pre-diabetes involved in regular SSG training for 12–16 weeks improved their maximal oxygen consumption (VO_2max_) by 10%–14% (de Sousa et al., 2014; de Sousa et al., 2017; Skoradal et al., 2018a). This finding is highly important since T2DM patients who improve their cardiorespiratory fitness by ∼5 mL/kg/min demonstrate a drop of cardiovascular mortality by all causes of 39%–70% (Church et al., 2005). Collectively, SSG football training represents an excellent intervention to improve glycaemic control, co-morbidity and performance of elderly T2DM patients.

## Cancer

Cancer is responsible for almost 18 million cases and 9 million deaths globally. It is worth noting that there is an increasing number of cancer survivors (Garcia and Thomson, 2014; Hashim et al., 2016). Although there is increased likelihood to overcome the disease, cancer survivors often need to deal with long-term debilitating health issues due to their treatment (Low et al., 2014). These issues include risk of recurrent cancer, CVD, osteoporosis, diabetes, chronic fatigue, and low quality of life (Elliott et al., 2011; Corner et al., 2013; Low et al., 2014). There is substantial amount of evidence that systematic exercise is beneficial for cancer patients and cancer survivors in respect to their daily function and quality of life (Stout et al., 2017). The American College of Sports Medicine (ACSM) recommends at least 150 min of moderate-intensity or 75 min of vigorous-intensity aerobic activity and 2-3 weekly sessions of strengthening exercise and stretching for cancer patients and if these recommendations cannot be achieved, then patients should avoid inactivity (Schmitz et al., 2010).

Prostate cancer (PCa) represents the most abundant non-cutaneous malignancy in men. Pca progress slowly and with available treatments such as androgen deprivation therapy (ADT) demonstrates 5–10-year survival rates (DeSantis et al., 2014; Gillessen et al., 2015). Although, ADT decreases blood testosterone levels markedly that ultimately decreases tumour growth and improves the effects of radiotherapy, it comes with some serious side effects such as sarcopenia, osteopenia and bone fractures, rise of fat mass and loss of physical function (DeSantis et al., 2014; Gillessen et al., 2015). Although exercise attenuates some side effects, most men with Pca are inactive before and after diagnosis (Karlsen et al., 2012).

Recreational football may offer a novel lifestyle intervention to increase physical activity levels in this population, and with two-three 1 h session weekly it is possible to full-fill the ACSM recommendations mentioned above with >75-min of vigorous-intensity aerobic training and 2-3 sessions of strength training (Krustrup et al., 2018a). Indeed, football training for men with Pca (mean age 67 years) was associated with a high attendance rate (46%–76%) despite being an intense activity with 25% of a training session having heart rates above 90% of maximum (Uth et al., 2014). In fact, locomotor field activity during a training session included more than 190 accelerations and around 300 decelerations at >0.6 m/s^2^ (Uth et al., 2016). These loads over a 12-week span were accompanied by a rise in lean body mass by 0.7 kg, lower limb strength by 6.7 kg, leg bone mineral content (BMC) by 14 g and whole-body BMC by 26 g as well as a marked rise of bone formation markers (i.e., P1NP and osteocalcin) (Uth et al., 2016). After 32 weeks of SSG training, marked favourable changes of bone mineral density was observed in both femoral shafts and total hips and these changes were associated with a sharp rise in stair climbing time and lower limb muscle power (Uth et al., 2016). In a nationwide 1-year implementation of this recreational football training concept, it was observed that the football training group (n = 105, mean age 68 years) had positive effects on fat percentage, bone mineralisation and mental health, along with a 40% lower number of hospitalisations in comparison with the control group (n = 109, mean age 69 years). (Bjerre et al., 2019). Interestingly, men who continued training during a 4.5 years follow-up retained these favourable bone adaptations whereas those who did not exhibited a marked loss of those training gains (Uth et al., 2018). Participation in recreational football training over a 5-year period was associated with a very high adherence rate rarely seen in exercise interventions in clinical settings (Mutrie et al., 2012). Moreover, a recent RCT intervention using recreational football training for women treated for stage I-III breast cancer have revealed that 1 year of 1-2 weekly 60 min session of football training is feasible and effective for middle-aged women and elderly women with positive effects on postural balance, leg muscle strength and lumbar spine bone mineral density (Uth et al., 2020; Uth et al., 2021) and no negative effects on breast cancer-specific upper-body morbidity, including lymphedema (Bloomquist et al., 2021).

In conclusion, SSG football training seems to offer a very promising approach to offset the deleterious side effects of ADT in men diagnosed with prostate cancer and may also be a feasible and effective approach for women after breast cancer.

## Effects on bone health

Deteriorated bone health is a pathophysiological condition characterized by decreased bone mineral density and elevated fracture risk and it is a major global health concern (Reginster and Burlet, 2006). Especially, because of the parallel increase in the elderly population and in sedentary behaviour worldwide (Kinsella and He, 2009), the prevalence of osteoporosis, osteopenia and bone fractures will increase markedly in the future (Cauley, 2013).

Human tissues, including the skeleton, experiences constant dynamic alterations, which are controlled by the remodelling process that comprises the coupled action of bone resorption by osteoclasts and bone formation by osteoblasts. During exercise training, mechanical forces are exerted on bones through ground reaction forces and/or through contractile forces exerted by the skeletal muscles (Steckling et al., 2016), which are supposed to induce osteogenic bone modelling. However, the osteogenic training stimulus appears to be activity specific.

Football is an intermittent and versatile high-intensity sporting activity characterized by multiple turns, jumps, and sprints, with accelerations and decelerations causing high rates of force application and large ground reaction forces (Uth et al., 2016; Krustrup et al., 2018a; Krustrup et al., 2018b), which is required in osteogenic training. Studies have shown remarkably high bone mineralisation in men and women with lifelong team sport, with leg BMD scores being higher than in 25–30-year-old untrained individuals (Hagman et al., 2018; Hagman et al., 2020). In a recent meta-analysis, evidence were provided that short- and medium-term football training interventions have marked osteogenic effects as indicated by changes in plasma bone turnover markers (Milanovic et al., 2022). Also, lower limb BMD is improved after SSG football training. This is supported by several randomised controlled trials on elderly and frail patient groups, showing marked improvement in bone mineralisation in the hip and lower spine (Helge et al., 2014; Bjerre et al., 2019).

Finally, postural balance and rapid muscle force which are considered to be important factors related to the risk of falls, are both affected positively by football participation (Krustrup et al., 2010b). Several cross-sectional studies comparing lifelong recreational and elite football players with untrained age matched 60–80-year-olds have shown that elderly footballs have an extraordinary good rapid muscle force and postural balance, to a level comparable to 30-year-old untrained men (Sundstrup et al., 2010; Helge et al., 2014; Randers et al., 2014). Moreover, several RCT intervention studies have shown that the postural balance is markedly improved with 2 times 60 min of football training for 3–12 months for 55–70-year-old women and for 60–80-year-old men (Andersen et al., 2016; Krustrup et al., 2017; Skoradal et al., 2018b; Bjerre et al., 2019). Thus, SSG football training appears to be a promising strategy to treat poor bone health and reduce the risk of falls and fractures in elderly. Walking football may also be an alternative to frail patient groups, since it has been shown to be feasible and safe for patients (Barbosa et al., 2020), but more research is warranted.

## Effect on biological aging

Aging is characterized as progressive decline of physiological functions and the increase in destructive processes in cells and organs, leading to increase susceptibility to death (López-Otín et al., 2013; Aunan et al., 2016; Barbosa et al., 2020). Deciphering the cellular mechanisms associated with the biology of aging are fascinating and complex. Several hallmarks of aging have been identified including, genomic instability, loss of proteostasis, epigenetic alterations, telomere attrition, mitochondrial dysfunction, cellular senescence, altered intercellular communication, deregulated nutrient-sensing and stem cell exhaustion (López-Otín et al., 2013). Among this telomere attrition and mitochondrial dysfunctions are the most common hallmarks of aging to study the impact of physical exercise on aging.

Telomeres are non-coding repetitive sequence that caps the chromosome end and maintain chromosome integrity but progressively shortens with each cell division, approximately 50–100 base pairs (Partride, 2010; Aunan et al., 2016). However, the rate of telomere shortening can be influenced by internal and external events. While negative lifestyle risk factors such as smoking and obesity are associated with shorter telomere length (Valdes et al., 2005), an active lifestyle displays a positive effect on telomere length as recently described (Nicholls, 2002; Hagman et al., 2021; Schellnegger et al., 2022). A recent systematic review and meta-analysis (Lim et al., 2022), has also shown the beneficial effects of exercise on mitochondrial health, where exercise intensity rather than modality was a better predictor of the positive effect. Somatic mitochondrial DNA damage and mutations are also part of the natural aging process, that contribute to impaired mitochondrial respiration (Trifunovic et al., 2004; Wallace, 2005), leading to structural and functional abnormalities in skeletal and myocardial tissues (Marín-García et al., 2001; Hoppel et al., 2009). Mitochondria are double-membranes organelles, which maintain the functional and structural integrity of pre- and post-mitotic cells, through involvement in cellular bioenergetics and the production of reactive oxygen species. Football training has shown a beneficial effect on preserving telomere length and increase in telomere stabilizing proteins compared to age-matched controls (Hagman et al., 2020; Hagman et al., 2021), suggesting the anti-aging effect in both men and women. In addition, our recent results showed that elite football and lifelong team handball training is associated with superior mitochondrial characteristics in women (Hagman et al., 2021).

The precise mechanisms on how recreational football training impacts aging at cellular level remain to be better elucidated. Physical activity and exercise positively modulate the skeletal muscle expression of markers involved in longevity pathways such as SIRT1 and SIRT3 in older rats (Ferrara et al., 2008; Palacios et al., 2009) and Heat Shock proteins (HSPs) (Murlasits et al., 2006). Further, activation of autophagy pathways positively affects physiological turnover of proteins, biological membranes, mitochondria and ribosome, avoiding damaged cellular components accumulation and protein aggregates, all aging hallmarks (Cuervo et al., 2005; Terman and Brunk, 2006; Schiavi and Ventura, 2014; Sakuma et al., 2017). Long-term football training increases skeletal muscle oxidation, stimulates musculoskeletal metabolic adaptations and improves cardiovascular system (Krustrup et al., 2010b; Schmidt et al., 2013; Alfieri et al., 2015; Bangsbo et al., 2015; Krustrup et al., 2018b; Mancini et al., 2020). Increased expression of muscle molecular markers involved in oxidative metabolism i.e., AMPKα1/α2, NAMPT, TFAM and PGC1α, MyHC β isoform expression and citrate synthase activity was higher in lifelong football-trained men than active untrained controls. These findings were associated with a healthier body composition and higher VO_2_max in trained compared to untrained elderly. Also, Erk1/2, Akt and FoxM1proteins expression, involved in the DNA repair and in senescence suppression pathway, were highest in skeletal muscle of lifelong veteran players compared to active untrained pairs (Mancini et al., 2017). Lifelong football training also affects the expression of most proteins and messengers involved in the auto-lysosomal and proteasome-mediated protein degradation (RAD23A, HSPB6, RAB1B, TRAP1, SIRT2), cellular growth and differentiation process (RPL4; RPL36; MRL37), proteasome promotion and autophagy processes (Bcl-2; HSP70; HSP90; ATG5-ATG12 protein complex) pathways in skeletal muscle of veterans compared to active untrained controls (Mancini et al., 2019; Nigro et al., 2020). SSG football training also affects the expression of c-miRNAs (miR-1303) involved in autophagy pathway and in cancer development (Cevenini et al., 2018; Mancini et al., 2021; Orlandella et al., 2021). These results contribute to in deep unravel the molecular and cellular effects mediated by lifelong football training as the most popular worldwide played sport on the longevity.

Despite the positive impact of SSG training on biological aging, this area is new and future research should aim to understand the long-term impact of recreational football training on biological aging and its molecular mechanisms. Future studies also need to investigate the tissues and cell type specific effect of football training on biological aging markers.

## Conclusion

Collectively, it is evident that regular small-sided game football training can be applied for the increasing elderly population as prevention and treatment of age-related non-communicable diseases and chronical conditions such as cardiovascular diseases, type 2 diabetes, sarcopenia, osteoporosis, and some types of cancer. Moreover, recreational football training is likely to have beneficial impact on biological aging and longevity.
